# Unveiling the unique gut microbial signatures in colorectal adenomas: establishment and validation of a cross-kingdom microbiome predictive model

**DOI:** 10.3389/fmicb.2026.1854806

**Published:** 2026-06-16

**Authors:** Bingfeng He, Zhiyong Xiao, Linfeng Zou, Jinmei Wei, Zhou Xiang, FangFang Sang, Xuefeng Guo

**Affiliations:** 1 Wuzhou Medical College, Wuzhou, China; 2 Department of General Surgery (Endoscopic Surgery), The Sixth Affiliated Hospital, Sun Yat-sen University, Guangzhou, China

**Keywords:** colorectal adenoma, cross-kingdom microbiome, gut microbiota, metagenome, predictive model

## Abstract

**Background:**

Colorectal adenoma (CA), the main precancerous lesion of colorectal cancer (CRC), originates in approximately 85–90% of CRC cases. With increasing demands for early diagnosis and treatment, gut microbiome research has become a forefront area. While numerous studies have shown that gut bacteria are closely related to the development of colorectal adenomas and cancer, research on viruses, archaea, and fungi is limited.

**Methods:**

From January 2019 to January 2024, this study collected 296 fecal samples from multiple centers and performed metagenomic analysis using shotgun sequencing. Principal coordinate analysis (PCoA) was conducted based on Bray-Curtis distance at the species level, α-diversity was calculated, and LEfSe analysis identified differential microorganisms. A random forest model was developed to distinguish adenoma patients from healthy individuals, with performance evaluated through internal validation using Bootstrap sampling and external validation with an independent cohort.

**Findings:**

Significant differences in the relative abundance of certain bacteria (e.g., *Phocaeicola_vulgatus* and *Prevotella_copri*), fungi (*Candida_albicans*), archaea (*Methanobrevibacter_oralis*), and viruses (*Streptococcus satellite phage Javan301*) were observed in adenoma patients. Spearman correlation analysis revealed complex network relationships among these microorganisms. The prediction model achieved a mean AUC of 0.80 ± 0.05 and an external validation AUC of 0.75, demonstrating stability and generalizability.

**Conclusion:**

This study shows significant cross-kingdom microbial signatures in colorectal adenoma patients, providing potential for developing new preventive and therapeutic methods. The predictive model, based on these differential microorganisms, exhibits robust and promising classification performance, offering potential for early adenoma detection.

## 1 Introduction

Colorectal cancer (CRC) holds a prominent position in global cancer incidence and mortality rates (Burnett-Hartman et al., 2021). Colorectal adenoma (CA), as a primary precancerous lesion of CRC, is indeed the origin of approximately 85–90% of all CRC cases (Bjerrum et al., 2020), with most cancers following the adenoma-to-carcinoma progression pathway, typically presenting as sporadic cases (Leslie, 2002). Given that many patients are diagnosed at an advanced stage of the disease, this underscores the critical importance of early screening and preventive measures (Ladabaum et al., 2020; Buskermolen et al., 2019).

In the context of early diagnosis and treatment of CA, research on the gut microbiome is emerging as a frontier area. Numerous studies have indicated that gut bacteria are closely related to the development and progression of colorectal adenomas and cancer, and can serve as biomarkers for early diagnosis (Zwezerijnen-Jiwa et al., 2023; Liu et al., 2021; Gao et al., 2020; Konishi et al., 2022). Ma et al. revealed the potential of microbiome-derived biomarkers as promising non-invasive tools for accurately detecting and distinguishing individuals with early-onset colorectal cancer (Kong et al., 2023). Chen et al. identified several metabolomic features of colorectal adenomas associated with certain gut microbes, which may predict future CRC (Kim et al., 2020). Despite these findings, most current research on the relationship between gut microbiota and diseases primarily focuses on bacteria, neglecting the roles of viruses, archaea, and fungi from other kingdoms.

The human gut microbiome is a complex ecosystem composed of microorganisms from multiple kingdoms, including prokaryotes (bacteria and archaea), viruses, and fungi. Yu Jun et al. demonstrated that cross-kingdom microbial features are associated with the efficacy of immune checkpoint inhibitors in various types of cancer, suggesting that cross-kingdom microbial biomarkers could be effective predictors of patient response to such therapies (Lin et al., 2024). Additionally, Naoyoshi Nagata et al. conducted shotgun metagenomic analyses of fecal samples from Crohn’s disease and ulcerative colitis patients and healthy controls, not only detailing bacterial species but also covering bacteriophages and eukaryotic microbes. This multi-kingdom analysis characterized the microbial ecology of these diseases and identified cross-kingdom interactions (Akiyama et al., 2024).

Although cross domain microbial features have been reported in these gastrointestinal diseases, the specific cross domain microbial features and their predictive potential in the adenoma stage have still been largely unexplored. Most existing biomarker studies focus on bacterial communities involved in carcinogenic processes, often overlooking early synergistic changes involving fungi, archaea, and viruses in precancerous lesions. The current research has not fully explored the potential impact of these microbial communities on the development of adenomas, especially their application as early diagnostic biomarkers. This study aims to comprehensively analyze cross disciplinary microbial communities including bacteria, viruses, archaea, and fungi, and explore their potential as biomarkers for developing predictive models.

## 2 Methods

### 2.1 Study design and sample collection

Microbiome analysis samples were obtained from residents in coastal and high-altitude regions. Inclusion criteria: aged 18–75 years and regularly attending outpatient examinations; no history of tumors. Exclusion criteria: (1) use of antibiotics (antibacterial or antifungal medications) within 3 months prior to colonoscopy, use of microbiological agents (such as probiotics and prebiotics) within 1 month, or use of any medications that could affect the gut microbiota or host immunity, such as hormones and immunosuppressants; (2) family history of familial adenomatous polyposis; (3) family history of inflammatory bowel disease; (4) acute infectious diseases; (5) HISTORY of gastrointestinal surgery; (6) incomplete colonoscopy or presence of high-risk factors for bleeding or perforation; (7) co-existing severe cardiovascular or pulmonary diseases.

Written informed consent was obtained from all participants prior to the study. Sample collection was conducted across multiple centers; high-altitude samples were obtained from large hospitals in Tibet and Qinghai, while coastal samples were collected from large hospitals in Guangdong and Fujian. Recruitment took place from January 1, 2019, to January 1, 2024.

Stool samples were collected prior to colonoscopy following strict standard operating procedures. Samples were immediately transferred into centrifuge tubes and stored at −80 °C until sequencing. Participants were grouped based on colonoscopy reports. The diagnosis of adenomas was based on a comprehensive evaluation of endoscopic findings, clinical data, and histopathological results.

The study protocol was approved by the Ethics Committee of the Sixth Affiliated Hospital of Sun Yat-sen University, and other participating centers also submitted their review results (Approval No.: 2021ZSLYEC-206). We confirm that ethical approval and permission for the dataset were obtained, and the study was conducted in accordance with the provisions of the Declaration of Helsinki (as revised in Fortaleza, Brazil, October 2013). The data obtained were collected and analyzed solely for this study; details are not publicly released, and strict information confidentiality rules were observed.

### 2.2 Metagenomic DNA isolation and sequencing

Following sample collection, bacterial DNA was isolated from 200 mg of stool using the QIAamp DNA Stool Mini Kit (Qiagen, Germany) according to the manufacturer’s instructions. DNA purity was assessed by measuring the optical density (OD) at 260/280 nm using a Nanodrop® spectrophotometer (IMPLEN, CA, USA), and DNA integrity was verified via 1% agarose gel electrophoresis. DNA concentration was quantified using the Qubit® dsDNA Assay Kit with a Qubit® 2.0 fluorometer (Life Technologies, USA).

### 2.3 Metagenomic sequencing and processing

DNA libraries were constructed using the NEBNext® Ultra DNA Kit and sequenced on the Illumina NovaSeq platform. Following sequencing, low-quality reads and host sequences were removed using fastp (Chen, 2023) and Bowtie2 (Zhou et al., 2024), yielding 1.61 Tb of high-quality data. The average number of clean reads per sample was approximately 21.8 million. After quality control with KneadData, taxonomic profiling was performed using Kraken2 (v2.0.7) against the standard RefSeq database (Li et al., 2025), and abundance estimation was conducted using Bracken (Li et al., 2023). Microbial community and metabolic pathway analyses were carried out using MetaPhlAn (v4.0.3) (Avershina et al., 2025) and HUMAnN (v3.6) (Franzosa et al., 2018), respectively, with pathways or species present in fewer than 10 samples being excluded. LEfSe (Khleborodova et al., 2024) analysis was employed to identify significantly different microbial taxa (*p* values and adjusted *p* values < 0.05), and the effect size was assessed via Linear Discriminant Analysis (LDA) (LDA score log10 > 2).

### 2.4 Microbiota diversity analysis

Microbial community diversity analysis was conducted using Python 3.6.5. The α-diversity indices, including the Shannon index, ACE index, Chao1 index, and Simpson index, as well as β-diversity metrics (Bray–Curtis distance and Jaccard similarity coefficient) were calculated. Principal coordinate analysis (PCoA) and permutational multivariate analysis of variance (PERMANOVA) were employed to assess the differences in microbial structures between different groups. Hierarchical clustering analysis was performed based on the average abundance of species.

### 2.5 Establishment and validation of predictive model

Based on the relative abundance of distinct gut microbiota, we employed the Random Forest (RF) algorithm, sourced from the scikit-learn package. To ensure the robustness of the model and address potential class imbalance issues, we implemented a Bootstrap resampling validation strategy, conducting 100 iterations.

In each iteration, a training subset is generated through sampling with replacement, while the out-of-bag (OOB) samples serve as the validation set. It is crucial to prevent information leakage by first independently fitting a StandardScaler on the training subset and then applying it to the validation subset. To address the issue of class imbalance, we applied the class_weight=‘balanced’ parameter when training the random forest classifier. This parameter automatically adjusts the weights to be inversely proportional to the class frequency in the input data.

In addition, a logistic regression (LR) model was established as a baseline for comparison. The model performance was evaluated using the area under the receiver operating characteristic curve (AUC). Based on the distribution of metrics across 100 bootstrap iterations, we calculated the 95% confidence intervals (95% CI) for AUC, sensitivity, and specificity using the percentile method. The confusion matrix metrics were calculated using a classification threshold of 0.5. Furthermore, we plotted a calibration curve to assess the consistency between predicted probabilities and actual outcomes.

### 2.6 Statistical analysis

The Mann–Whitney *U* test was employed to verify the abundance differences in metagenomic analysis data between the adenoma group and the healthy control group. For each species in the abundance table, its relative abundance values in the two groups were extracted and subjected to a two-sided test. All *p* values were corrected for multiple testing using the Benjamini–Hochberg method to calculate the False Discovery Rate (FDR) *Q* value, with *Q* < 0.05 considered statistically significant. The analysis process was automated using a script in Python 3.6.5.

## 3 Results

### 3.1 Study characteristics

We collected a total of 296 samples for metagenomic sequencing. Detailed sequencing depth and quality metrics can be found in Supplementary Table S1. For the construction and internal validation of the predictive model, 184 samples were from the coastal region, including 106 adenoma patients (average age 55 years; 57.55% male) and 78 healthy controls (average age 51 years; 35.90% male). For the external validation of the model, 112 samples were from the plateau region, comprising 44 adenoma patients (average age 52 years; 65.91% male) and 68 healthy controls (average age 51 years; 64.71% male). Detailed baseline data are provided in Supplementary Table S2. All fecal samples were processed using the same protocol to minimize batch effects caused by technical factors.

### 3.2 Cross-kingdom microbial characteristics differ between colorectal adenoma patients and healthy individuals

**Figure 1:**
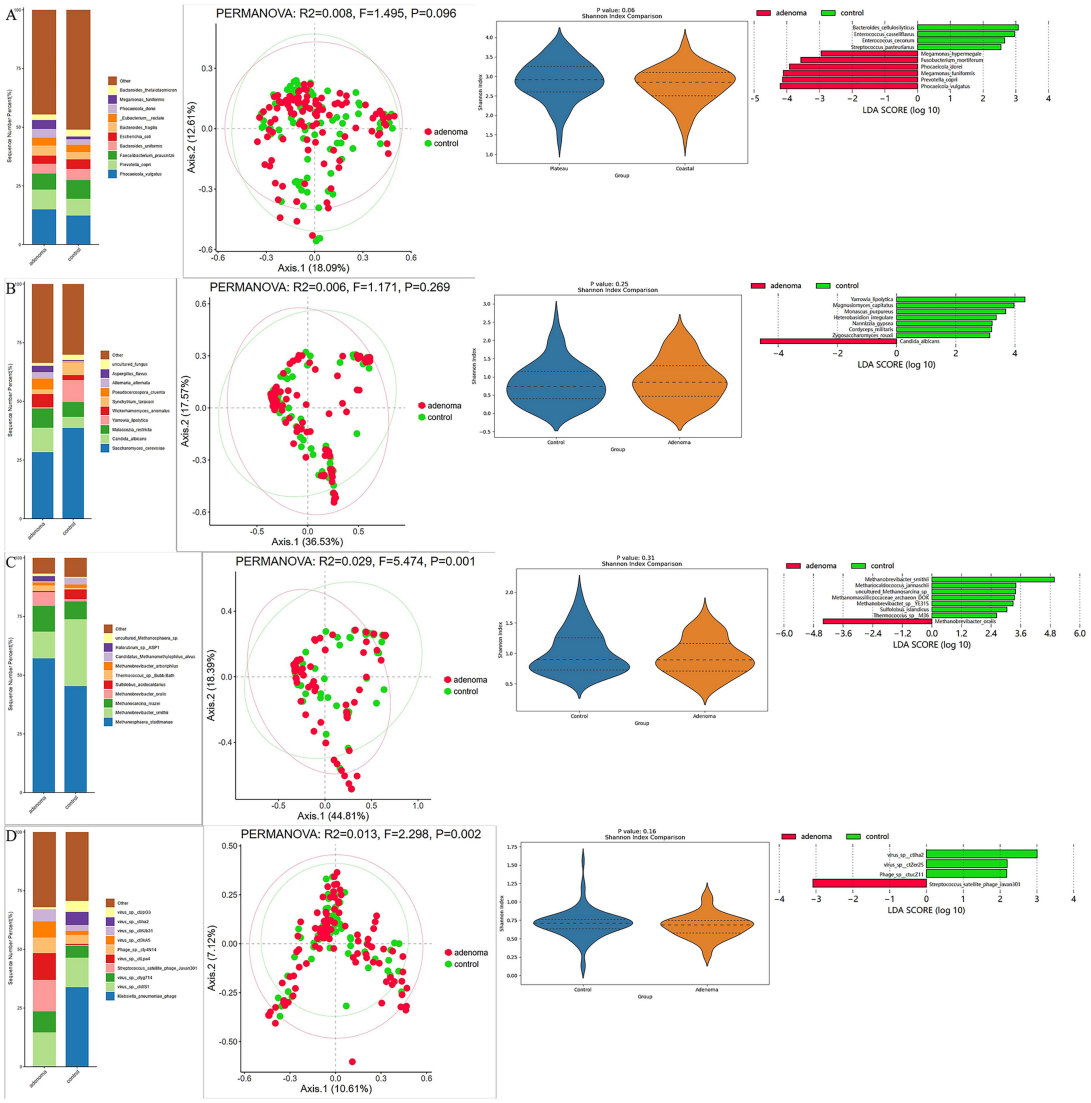
Descriptive analysis of 184 coastal population samples across kingdoms. From left to right, the figures show the proportion of microbial species within each population, visualized using principal coordinate analysis (PCoA) based on Bray–Curtis distance of species-level composition, violin plots depicting α-diversity based on relative abundance, and LEfSe (linear discriminant analysis effect size) differential analysis based on species-level relative abundance. **(A)** Descriptive analysis of bacteria. **(B)** Descriptive analysis of fungi. **(C)** Descriptive analysis of archaea. **(D)** Descriptive analysis of viruses.

We observed that the most abundant bacterial species in both healthy individuals and colorectal adenoma patients was *Phocaeicola_vulgatus*. PERMANOVA analysis did not reveal statistically significant differences in the overall bacterial community structure between the colorectal adenoma group and the control group (*R*^2^ = 0.008, *F* = 1.495, *p* = 0.096). Similarly, regarding α-diversity, the difference in the Shannon index between the two groups did not reach statistical significance (*p* value = 0.06), suggesting that the overall bacterial diversity remains relatively stable during the adenoma stage. Despite the lack of significant global structural separation, LEfSe analysis identified specific bacterial species enriched in the colorectal adenoma group, such as *Phocaeicola_vulgatus* and *Prevotella_copri*. To ensure the robustness of these findings and address potential sensitivities of LEfSe to compositionality, we further validated these differential taxa using the Mann–Whitney *U* test with Benjamini–Hochberg FDR correction. These key species (including *P. vulgatus* and *P. copri*) remained statistically significant in the independent validation (FDR-adjusted *p* < 0.05), confirming their potential roles in the development of adenomas (Supplementary Table S3, Figure 1A).

At the fungal level, the most abundant species in both groups was *Saccharomyces_cerevisiae*. PERMANOVA did not show significant differences in fungal community structure between the two groups (*R*^2^ = 0.006, *F* = 1.171, *p* = 0.269), suggesting similar fungal community structures in colorectal adenoma and control groups. There was also no significant difference in Shannon index between the two groups (*p* value = 0.25), further supporting the stability of fungal diversity. Additional fungal α-diversity metrics are shown in Supplementary Figure S1B. Although there were no overall significant differences in fungal communities, LEfSe analysis identified *Candida_albicans* as relatively abundant in the adenoma group (Figure 1B). Consistent with our bacterial analysis, this finding was corroborated by the Mann–Whitney *U* test, where *Candida_albicans* showed a significant difference after FDR correction (Supplementary Table S3), supporting its potential association with colorectal adenoma.

At the archaeal level, the most abundant species in both groups was *Methanosphaera_stadtmanae*. PERMANOVA results indicated significant differences in archaeal community structure between the colorectal adenoma group and the control group (*R*^2^ = 0.029, *F* = 5.474, *p* = 0.001), suggesting that archaeal communities might play a role in adenoma development. There was no significant difference in Shannon index between the two groups (*p* value = 0.31), indicating consistent archaeal diversity. Additional metrics are shown in Supplementary Figure S1C. LEfSe analysis revealed that *Methanobrevibacter_oralis* was enriched in the colorectal adenoma group. This enrichment was further confirmed by the Mann–Whitney U test (FDR-adjusted *p* < 0.05), suggesting that *Methanobrevibacter_oralis* a robust biomarker potentially associated with adenoma progression (Supplementary Table S3, Figure 1C).

At the viral level, the most abundant virus in adenoma patients was *virus_*sp.*_ctdtS1*, while in the control group, it was *Klebsiella_pneumoniae_phage*. PERMANOVA results showed significant differences in viral community structure between the colorectal adenoma group and the control group (*R*^2^ = 0.013, *F* = 2.298, *p* = 0.002), suggesting that viral communities might be important in adenoma development. No significant differences were found in *α*-diversity metrics between the two groups (Supplementary Figure S1D), indicating no obvious changes in viral diversity. LEfSe analysis revealed an enrichment of *Streptococcus satellite phage Javan301* in colorectal adenoma patients. This differential abundance was validated using the Mann–Whitney U test with FDR correction (Supplementary Table S3). Given that *Streptococcus satellite phage Javan301*is a specific bacteriophage infecting Streptococcus, we speculate that it might indirectly promote the occurrence and development of colorectal adenomas by modulating the activity or community structure of its host bacteria (Figure 1D).

### 3.3 Correlations among enriched microorganisms across kingdoms in adenoma patients

**Figure 2:**
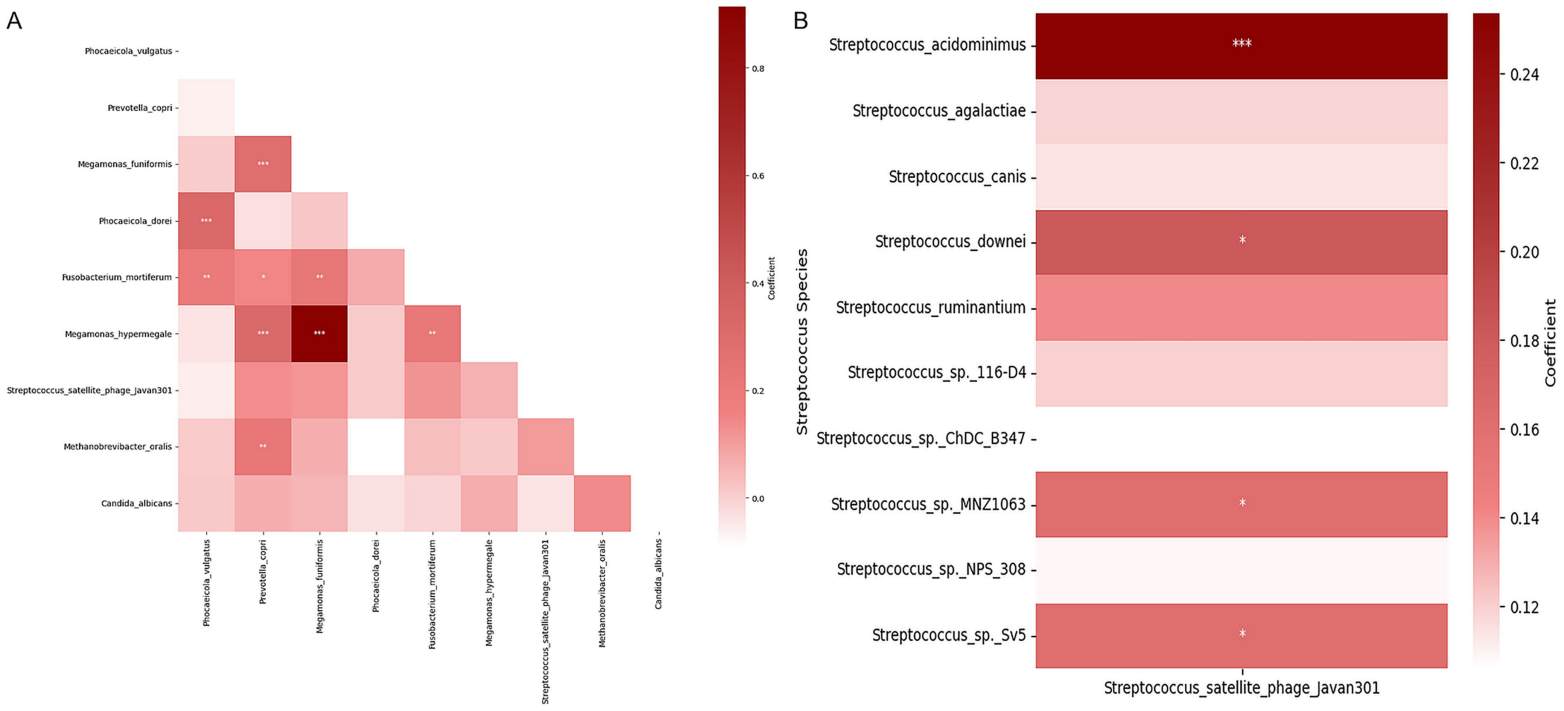
Spearman correlation analysis; significance analysis using Fisher’s test. **(A)** Correlation analysis among the four kingdoms of microorganisms. **(B)** Correlation analysis between *Streptococcus* phage Javan301 and various *Streptococcus* species. Stars indicate the level of significance: ^*^p << 0.05; ^**^p << 0.01; ^***^p << 0.001.

To further explore the relationships among these differential microorganisms, we performed Spearman correlation analysis based on their relative abundances. The analysis revealed several significant positive correlations between different bacterial species. Specifically: *Prevotella_copri* and *Megamonas_hypermegale* (*R* = 0.315; *p* value <0.001); *Phocaeicola_vulgatus* and *Phocaeicola_dorei* (*R* = 0.318; *p* value <0.001); *Megamonas_funiformis* and *Megamonas_hypermegale* (*R* = 0.913; *p* value <0.001). These correlations suggest potential co-occurrence among these bacterial populations that collectively influence the structure and function of the microbiome. We also found a positive correlation between the archaeon *Methanobrevibacter_oralis* and *Prevotella_copri* (*R* = 0.219; *p* value <0.01), this co-occurrence pattern suggests that these two taxa may share similar ecological niches. For instance, hydrogen produced by *Prevotella_copri* during polysaccharide degradation can be used by *Methanobrevibacter_oralis* for methane production, which not only reduces hydrogen pressure in the gut but may also promote the growth of *Prevotella* species. However, no statistically significant correlations were observed between fungi, viruses, and other kingdoms of microorganisms (Figure 2A; Supplementary Table S5).

Given the notable enrichment of the bacteriophage *Streptococcus* satellite phage Javan301 identified in adenoma patients, we conducted a correlation analysis between this phage and its host bacteria. The results showed significant positive correlations between *Streptococcus* satellite phage Javan301 and various streptococcal species: *Streptococcus acidominimus* had the highest correlation coefficient with *Streptococcus* satellite phage Javan301, reaching 0.253, with extremely high statistical significance (*p* value <0.001), indicating a very strong positive correlation. *Streptococcus downei*, Streptococcus sp._MNZ1063, and *Streptococcus* sp._SV5: These streptococcal species had correlation coefficients with *Streptococcus* satellite phage Javan301 of 0.181, 0.160, and 0.161, respectively, all achieving statistical significance (*p* value <0.05). These results indicate a statistically significant but weak correlation between these streptococcal species and the phage. Other streptococcal species such as *Streptococcus agalactiae*, *Streptococcus canis*, Streptococcus ruminantium, *Streptococcus* sp.*_116-D4*, *Streptococcus* sp.*_ChDC_B347*, and *Streptococcus* sp*._NPS_308* showed weaker or non-significant correlations with Streptococcus satellite phage Javan301 (Figure 2B; Supplementary Table S6).

These findings reveal a complex network of co-occurrence between bacteriophages and their potential host bacteria. Particularly for those streptococcal species showing significant positive correlations, this might indicate higher infection rates of Streptococcus satellite phage *Javan301* within these bacterial populations or that the presence of the phage promotes bacterial growth and proliferation. This positive correlation may reflect potential host dependency or synchronized population dynamics.

### 3.4 Adenoma prediction model based on differential microorganisms

Given the abundant cross-domain microorganisms identified in patients with adenoma, we employed a binary classification approach to investigate the utility of these microorganisms in predicting the occurrence of adenoma. We compared the performance of a Random Forest (RF) model with that of a Logistic Regression (LR) baseline model.

**Figure 3:**
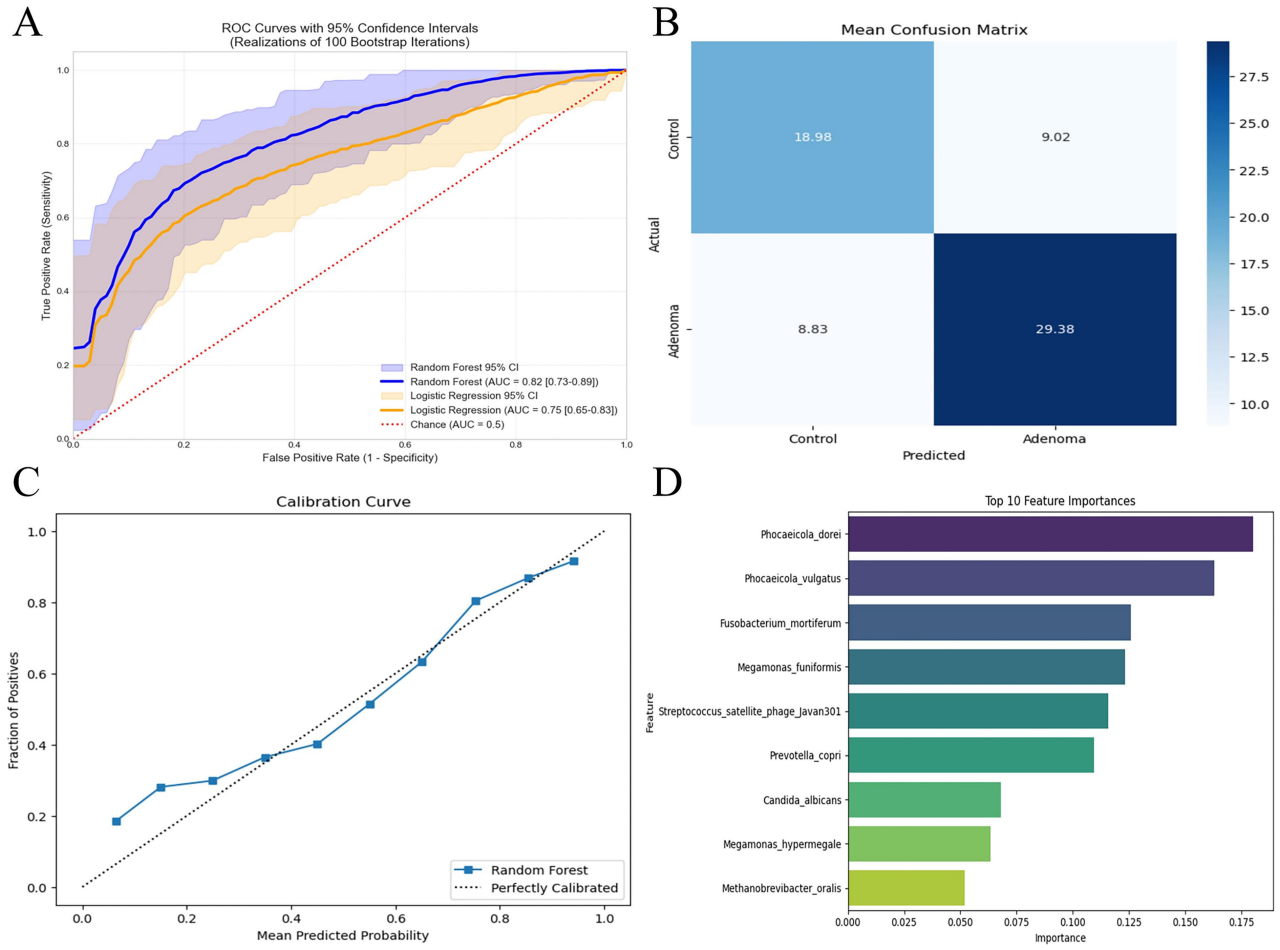
Prediction model for colorectal adenoma based on 9 transkingdom microorganisms. **(A)** The mean ROC curve showing the performance of the prediction model. The mean ROC AUC is 0.80 ± 0.05; **(B)** The average confusion matrix indicating the classification performance of the model. This matrix shows the true and predicted labels for the negative and positive classes; **(C)** Calibration curves demonstrating the agreement between the predicted probabilities and the actual outcomes; **(D)** a bar plot illustrating the average feature importance of the 9 microorganisms used in the prediction model.

As shown in Figure 3A, compared to the LR model, the RF model exhibits stronger discriminative ability. The AUC of the RF model is 0.82 (95% confidence interval: 0.73–0.89), whereas the AUC of the logistic regression model is 0.75 (95% confidence interval: 0.65–0.83). The shaded area in Figure 3A represents the 95% confidence interval obtained through 100 bootstrap iterations, indicating the stability of the RF model’s performance.

Based on the confusion matrix analysis with a threshold of 0.5, the sensitivity of the final RF model is 0.77, and the specificity is 0.68. The model also exhibits a positive predictive value (PPV) of 0.76 and a negative predictive value (NPV) of 0.68. The calibration curve demonstrates good consistency between predicted probabilities and actual observed results, confirming the reliability of the model (Figure 3C).

In terms of feature importance, the microorganisms that contributed the most to prediction were identified as *Phocaeicola dorei*, *Phocaeicola vulgatus*, and *Fusobacterium mortiferum* (Figure 3D).

### 3.5 Cross-kingdom microbial characteristics differ between populations at different altitudes

**Figure 4:**
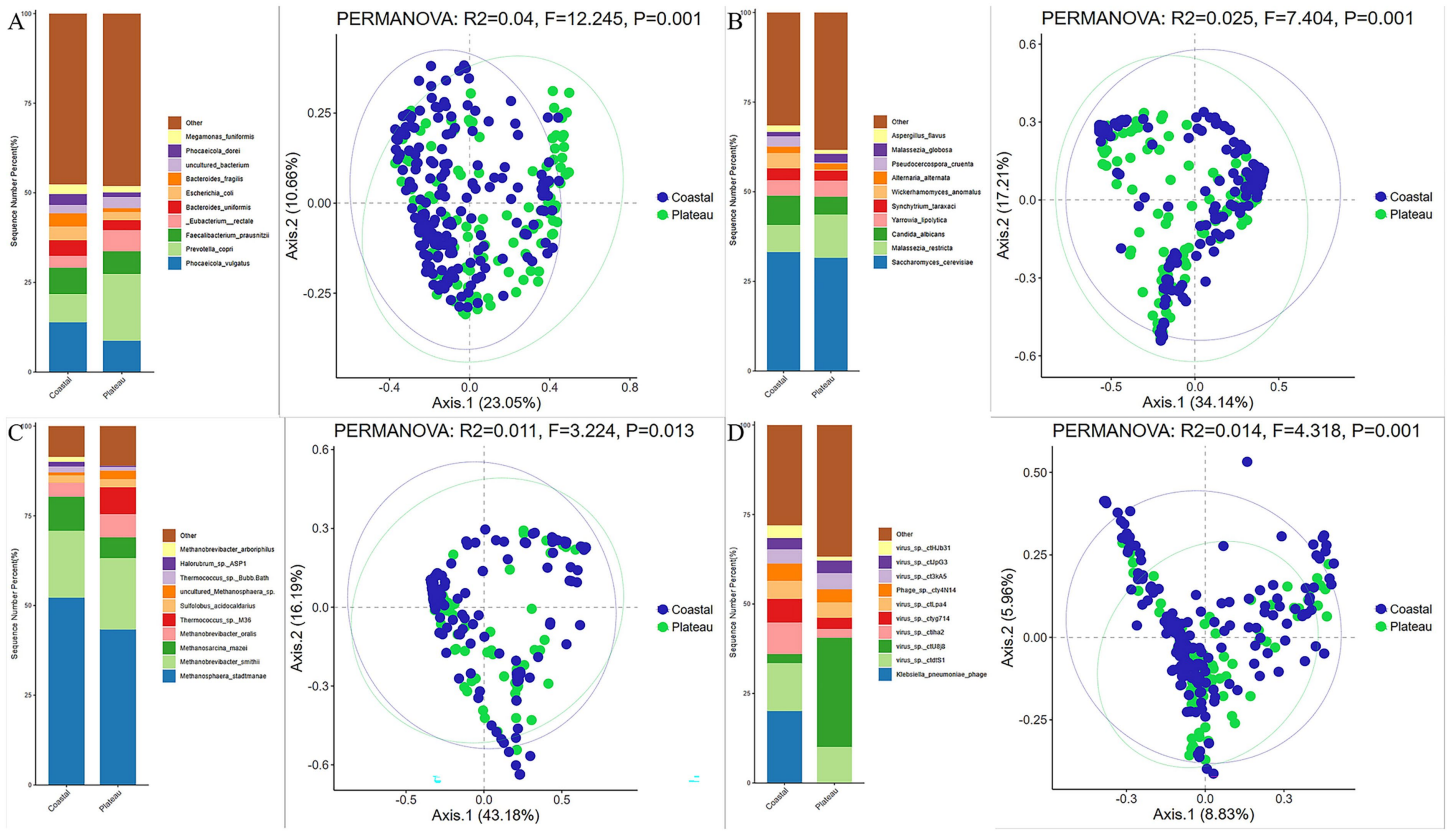
Descriptive analysis of 184 coastal population and 112 plateau population samples across kingdoms. From left to right, the figures show the proportion of microbial species within each population, visualized using principal coordinate analysis (PCoA) based on Bray–Curtis distance of species-level composition. **(A)** Descriptive analysis of bacteria. **(B)** Descriptive analysis of fungi. **(C)** Descriptive analysis of archaea. **(D)** Descriptive analysis of viruses.

Given that we included two distinct populations—those residing in plateau regions and those in coastal regions—we further analyzed whether the place of residence impacts microbial structure. The results showed that at the bacterial level, the most abundant species in the coastal population was *Phocaeicola_vulgatus*, while in the plateau population, it was *Prevotella_copri*. PERMANOVA analysis revealed significant differences in bacterial community structure between the plateau and coastal populations (*R*^2^ = 0.04, *F* = 12.245, *p* = 0.001) (Figure 4A), validating the “background signal.” However, despite this distinct microbial background, *Phocaeicola vulgatus* and *Megamonas_funiformis* were still identified as key species enriched in the plateau adenoma group. Mann–Whitney *U* tests with FDR correction confirmed that these species were significantly different in the plateau cohort, suggesting they are robust biomarkers for adenomas independent of geography (Supplementary Figure S2A, Supplementary Table S4). At the fungal species level, both populations had *Saccharomyces_cerevisiae* as the most abundant species. PERMANOVA indicated significant differences in fungal community structure between the plateau and coastal populations (*R*^2^ = 0.025, *F* = 7.404, *p* = 0.001) (Figure 4B). Similarly, while the overall fungal structure differed between regions, C*andida_albicans* was consistently enriched in adenoma patients in both the coastal and plateau cohorts. This consistency was statistically validated (FDR-adjusted *p* < 0.05), indicating that the enrichment of *Candida_albicans* specifically associated with adenoma development rather than the high-altitude environment (Supplementary Figure S2B, Supplementary Table S4). At the archaeal species level, both populations had *Methanosphaera_stadtmanae* as the most abundant species. PERMANOVA showed significant differences in archaeal community structure between the plateau and coastal populations (*R*^2^ = 0.01, *F* = 3.244, *p* = 0.013), indicating significant differences (Figure 4C). *Methanobrevibacter_oralis* was identified as a significant biomarker in the plateau adenoma group. The validation of this taxon in the plateau cohort confirms that archaeal differences are linked to adenoma pathology (Supplementary Figure S2C, Supplementary Table S4). At the viral level, the most abundant species in the coastal population was *Klebsiella_pneumoniae_phage*, while in the plateau population, it was *virus_*sp*._ctdtS1*. PERMANOVA revealed significant differences in viral community structure between the plateau and coastal populations (*R*^2^ = 0.014, *F* = 4.318, *p* = 0.001), indicating a clear trend of difference (Figure 4D). Nevertheless, *Streptococcus* satellite phage Javan301 was identified as a key differential taxon in the plateau adenoma group. This finding, validated by the Mann–Whitney *U* test, suggests that the phage-host co-occurrence involving *Streptococcus* are a consistent feature of adenoma development, regardless of the geographic location (Supplementary Figure S2D, Supplementary Table S4).

**Figure 5:**
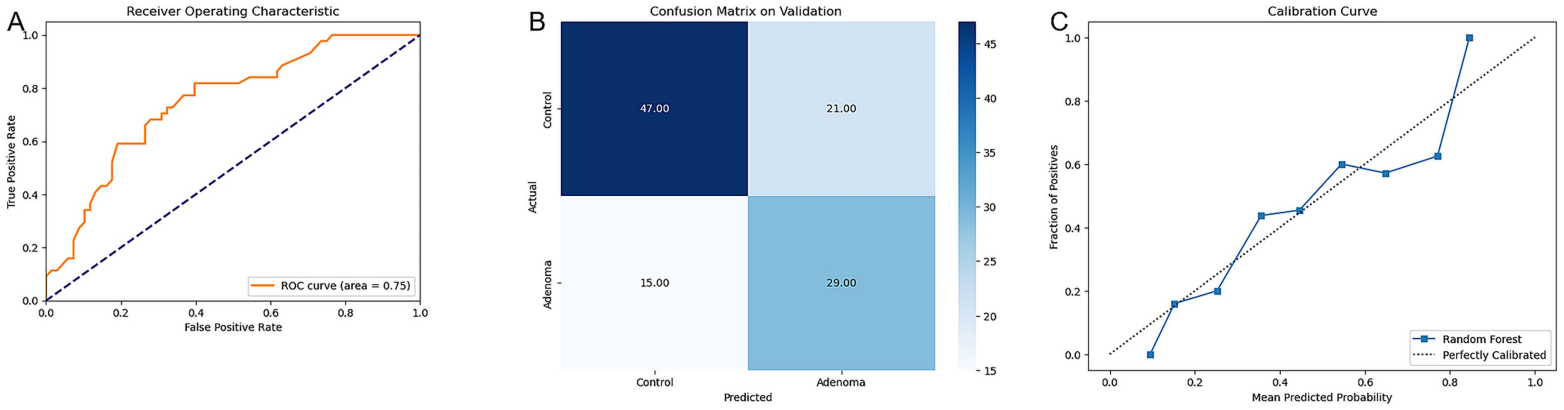
External validation performance of the prediction model. **(A)** The ROC curve showing the performance of the prediction model. The AUC is 0.75; **(B)** the confusion matrix indicating the classification performance of the model. This matrix shows the true and predicted labels for the negative and positive classes; **(C)** calibration curves demonstrating the agreement between the predicted probabilities and the actual outcomes.

Subsequently, we used samples from the plateau population to perform external validation of the predictive model, aiming to demonstrate its generalizability and robustness. The results showed that the external validation AUC reached 0.75 (Figure 5A). Sensitivity was 0.58, specificity was 0.76, PPV was 0.66, NPV was 0.69, and the F1 score was 0.62 (Figure 5B). Calibration curves also demonstrated the robustness of the model (Figure 5C). Despite the significant differences in microbial community structures between populations at different altitudes, our model still exhibited good performance, further validating the value of these cross-kingdom microbial signatures for predicting colorectal adenomas.

This analysis underscores the impact of geographic location on microbial composition and highlights the importance of considering these variations when developing diagnostic tools and therapeutic strategies. The consistent performance of the prediction model across different populations suggests its potential utility in diverse settings, reinforcing the significance of cross-kingdom microbial biomarkers in colorectal adenoma detection and prevention.

## 4 Discussion

Alterations in the gut ecosystem are associated with changes in human health and disease, including colorectal adenomas (Burnett-Hartman et al., 2021). Fecal metagenomics serves as a valuable tool for quantifying the gut microbiome and holds potential diagnostic value (Zeller et al., 2014). Notably, *F. nucleatum* has been linked to CRC (Castellarin et al., 2012; Kostic et al., 2012), with its mechanisms clarified in mouse models (Yang et al., 2017). However, the human gut microbiota comprises not only bacteria but also viruses, protozoa, and fungi, all of which are essential for human health (Barrera-Vazquez and Gomez-Verjan, 2020). Current research primarily focuses on bacteria within the gut ecosystem (Yachida et al., 2019; Coker et al., 2022), while the complex associations between other types of microorganisms—particularly fungi, viruses, and archaea—and human health remain largely unexplored. This may be due to their relatively low abundance in the human gut. This low abundance similarly affects the study of non-bacterial microorganisms, as a single metagenomic dataset may not provide sufficient sequencing depth and read counts for effective profiling of these rare microbes. Therefore, comprehensive metagenomic analysis could offer an alternative approach to understanding colorectal adenoma development through relevant changes in the gut environment.

In this study, to uncover cross-kingdom microbial changes in bacteria, fungi, viruses, and archaea related to colorectal adenomas, we analyzed fecal metagenomic sequencing results from 296 individuals across different regions with colorectal adenomas and control subjects. To date, the relationship between gut bacteria and colorectal adenomas has been extensively studied, but the roles of eukaryotes, archaea, and viruses have often been overlooked. Moreover, by integrating comparable studies, multi-cohort studies can enhance statistical power and reduce cohort-specific biases (Pereira et al., 2018). In our study, metagenomic sequencing enabled us to depict the overall impact of the gut microbiota in colorectal adenomas, revealing macro and micro differences in cross-kingdom gut microbes such as bacteria and eukaryotes between colorectal adenoma and healthy control populations (Figure 1).

Firstly, we investigated compositional changes in the gut microbiota at the species level in colorectal adenoma patients compared to healthy controls. Among community characteristics across different kingdoms, we observed significant bacterial and archaeal community differences between adenoma patients and healthy controls, whereas fungal and viral community differences were relatively minor. In the bacterial kingdom, LEfSe analysis identified enrichment of the bacterial species *Prevotella_copri* in the adenoma group. Studies using the gutMEGA database screened candidate bacteria that might serve as non-invasive biomarkers for colorectal cancer (Yao et al., 2021), including *Prevotella_copri*, aligning with our findings, suggesting its involvement in adenoma development and progression. In fungal and viral communities, although overall diversity did not show significant differences, some specific microbial enrichments were observed. For example, enrichment of *Candida_albicans* in the adenoma group may be related to the development of colorectal adenomas. In certain environments, particularly in immunocompromised systems, fungi can conditionally become pathogenic. This finding is consistent with previous research on the potential pathogenic role of *Candida_albicans* in gut diseases. Candida fungi can translocate to the lamina propria of the gut and exacerbate colitis (Li et al., 2022), and promote colorectal adenomas (Zhang et al., 2024). *Candida_albicans* may promote colorectal adenomas by inducing glycolysis in macrophages and enhancing the secretion of IL-22 by innate lymphoid cells (Zhu et al., 2021).

Although archaea are stable components of the gut microbiota, they are often overlooked in studies of host-associated microbial communities in CRC (Wong and Yu, 2019). Archaea possess distinct characteristics from bacteria and eukaryotes, including unique flagellins and the absence of peptidoglycan and endotoxins in their cell walls (Zillig, 1991; Kandler and König, 1998). Despite their relatively lower abundance compared to bacteria in the gut, archaea are crucial for maintaining the homeostasis of the host’s gut microenvironment. They participate in methanogenesis, carbohydrate metabolism, trimethylamine (TMA) metabolism, and immune regulation (Brugère et al., 2014; Borrel et al., 2020). Similarly, our study found significant enrichment of *Methanobrevibacter_oralis* in colorectal adenoma populations. *Methanobrevibacter* is a Gram-negative bacterium and a methanogen, playing a critical role in methanogenesis and potentially affecting disease-related processes (Samuel et al., 2007). Animal experiments have confirmed a causal relationship between methane production and slowed gut transit (Pimentel et al., 2006), while human studies have observed associations between baseline breath methane levels and constipation (Singh et al., 2020), a risk factor for CRC development, suggesting the potential involvement of high methanogens in CRC (Wang et al., 2023). *Methanobrevibacter_oralis* may be associated with various metabolic and gastrointestinal diseases (Samuel et al., 2007). Its widespread colonization in the human gut and ability to evade the host immune system underscore its potential as a pathogenic factor in CRC. Furthermore, *Methanobrevibacter* has been detected in multiple body cavities and is associated with inflammatory diseases (Sogodogo et al., 2019; Drancourt et al., 2021), highlighting its translocation capability and potential role as a pathogenic factor in CRC.

Additionally, viral community analysis revealed enrichment of Streptococcus satellite phage Javan301 in the adenoma group, which may indirectly promote colorectal adenoma development by modulating the host bacterial community structure. Streptococcus satellite phage Javan301 is a satellite phage infecting *Streptococcus* species (Zhao et al., 2025).

Notably, previous studies have indicated that the synergistic interactions between certain fungi and bacteria in the gut are closely related to human diseases. For instance, Hoarau et al. (2016) found a positive correlation between *Candida tropicalis* and two bacterial species—*Serratia marcescens* and *Escherichia coli*—in Crohn’s disease patients. The interaction among these three microorganisms can promote the formation of robust biofilms, which may lead to host tissue damage and specific immune responses (Cavalcanti et al., 2015). Therefore, interactions between gut fungi and bacteria play a crucial role in shaping the gut microbiota ecosystem (Sokol et al., 2017; Richard and Sokol, 2019). To further explore microbial interactions in adenoma patients, we conducted Spearman correlation analysis. Our study revealed a significant positive correlation between archaeon *Methanobrevibacter_oralis* and *Prevotella_copri* (*R* = 0.219, *p* value <0.01). This positive correlation suggests that these two microorganisms potentially co-occur due to shared ecological niches. While previous literature suggests a metabolic syntrophy where *Prevotella_copri* produces hydrogen during polysaccharide degradation, which is utilized by *Methanobrevibacter_oralis* for methanogenesis, our data primarily confirm the co-occurrence of these taxa in the adenoma cohort.

Moreover, probiotics capable of producing short-chain fatty acids (SCFAs) are essential for colon health but are often depleted in CRC patients. Importantly, methanogens play a critical role in shifting the metabolic profile of the microbiota towards optimal SCFA production by consuming end products of bacterial metabolism and preventing metabolite accumulation, which is crucial for colonic homeostasis (Li et al., 2025).

Furthermore, based on the notable enrichment of the bacteriophage Streptococcus satellite phage Javan301 identified in adenoma patients, we further analyzed the correlation between this phage and its host bacteria. The results showed significant positive correlations between Streptococcus satellite phage Javan301 and various streptococcal species (*p* value <0.001). This strong correlation likely reflects close interactions between the phage and its host bacteria. Typical interactions between phages and their host bacteria are highly specific and often closely tied to individual bacterial strains. Phage-bacterial interaction networks exhibit nested and modular structures, with these interactions evolving dynamically, influenced by the localization of host bacteria in different organs and tissues and the complexity of interaction networks (Flores et al., 2011). Phage activity significantly impacts the quantity and behavior of host bacteria and mediates gene transfer among bacteria during host inflammation. Phages are closely related to the balance and imbalance of the microbiota, influencing human health through direct predation of bacteria or indirect pathways such as affecting metabolism and the immune system (Petrovic Fabijan et al., 2020). Phages replicate using the metabolic machinery of host bacteria, while host bacteria may gain adaptive advantages from the presence of phages, such as enhanced growth capabilities or metabolic efficiency (Luo et al., 2023). Although our correlation analysis suggests potential ecological relationships, these can only serve as hypotheses for future mechanism validation.

Additionally, the composition and function of the gut microbiota are influenced by various factors, including diet, lifestyle, and environmental exposure. These factors can alter the structure of the microbial community, thereby affecting the occurrence and development of colorectal adenomas. Therefore, we speculate that differences in microbial communities among populations at different altitudes may be related to the pathogenesis of colorectal adenomas, but specific mechanisms and causal relationships require further detailed analysis and validation.

Microbiome-related biomarkers have significant potential applications in distinguishing between different stages of colorectal adenomas (Samuel and Gordon, 2006). For instance, researchers led by Kumpitsch et al. (2021) constructed a predictive model based on 10 key bacterial species, with *Fusobacterium nucleatum* showing the best performance in distinguishing between adenoma and colorectal cancer patients. Additionally, studies have shown that precancerous lesions of colon cancer are closely related to changes in the abundance of *Faecalibacterium* species (Bier et al., 2018). These findings partially reveal the potential utility of gut microbiota in distinguishing colorectal adenoma patients from healthy controls.

In this study, we used a population from coastal regions as the training set and internal validation set to build a model based on differential cross-kingdom microbial communities for identifying colorectal adenoma patients versus healthy controls. This approach provides a potential tool for the early detection of colorectal adenomas. The developed Random Forest (RF) model achieved an average AUC of 0.80 ± 0.05. To further assess the external validity of the model, we validated it using a population from plateau regions, achieving an AUC of 0.75. Demonstrating the model’s generalizability across distinct geographical populations. However, we objectively note that the sensitivity in the external validation cohort was 0.58, which is lower than the internal validation results. This performance gap reflects the challenges inherent in real-world applications, particularly when extrapolating from the coastal population to the plateau population.

Despite these advantages, there are still several limitations worth considering. Firstly, we adopted strict exclusion criteria, but this retrospective study relied on participants’ self-reported health questionnaires, and we lacked detailed quantitative data on dietary patterns. As we all know, these factors can significantly modulate gut microbiota, and incomplete recording may introduce potential confounding effects.

Secondly, although our study successfully validated the model across different geographic populations, ranging from coastal areas in Guangdong to high-altitude regions in Xizang and Qinghai, the specific impact of regional dietary habits on adenoma-specific microbial characteristics still needs to be further explored in larger prospective cohort studies. Future research will incorporate standardized dietary diaries to refine these predictive models.

Furthermore, due to the nature of initial screening cohorts, detailed histopathological stratifications were not fully recorded in this study, which limits the stratification analysis of microbial signatures across different adenoma severities.

The similar predictive capabilities of models based on the same cross-kingdom microbial biomarkers across different cohorts preliminarily demonstrate the robustness and generalizability of the model. However, to further confirm its broad applicability, larger cohort studies are needed for verification. This finding underscores the importance of gut microbiota as diagnostic tools and provides a basis for their application in future clinical practice.

## Data Availability

The datasets presented in this study can be found in online repositories. The names of the repository/repositories and accession number(s) can be found at: https://www.ncbi.nlm.nih.gov/, PRJNA1194680 and PRJNA1194747. The processed microbiome data and the Python code used for the statistical analysis and model evaluation in this study are available in the GitHub repository: https://github.com/HBF-SMALL/Adenoma-prediction-model.git.

## Supplementary material

SUPPLEMENTARY FIGURE S1 **(A)** Violin plots of α-diversity at the bacterial species level. **(B)** Violin plots of α-diversity at the fungal species level. **(C)** Violin plots of α-diversity at the archaeal species level. **(D)** Violin plots of α-diversity at the viral species level. Statistical differences were compared using the Mann-Whitney *U* test.

SUPPLEMENTARY FIGURE S2 Lefse analysis of 112 high-altitude population samples: **(A)** Lefse analysis of bacteria; **(B)** Lefse analysis of fungi; **(C)** Lefse analysis of archaea; **(D)** Lefse analysis of viruses.

## References

[ref1] Akiyama S. Nishijima S. Kojima Y. Kimura M. Ohsugi M. Ueki K. . (2024). Multi-biome analysis identifies distinct gut microbial signatures and their crosstalk in ulcerative colitis and Crohn's disease. Nat. Commun. 15:10291. doi: 10.1038/s41467-024-54797-8, 39604394 PMC11603027

[ref2] Avershina E. Qureshi A. I. Winther-Larsen H. C. Rounge T. B. (2025). Challenges in capturing the mycobiome from shotgun metagenome data: lack of software and databases. Microbiome 13:66. doi: 10.1186/s40168-025-02048-3, 40055808 PMC11887097

[ref3] Barrera-Vazquez O. S. Gomez-Verjan J. C. (2020). The unexplored world of human virome, mycobiome, and archaeome in aging. J. Gerontol. A Biol. Sci. Med. Sci. 75, 1834–1837. doi: 10.1093/gerona/glz274, 31802114

[ref4] Bier A. Braun T. Khasbab R. di Segni A. Grossman E. Haberman Y. . (2018). A high salt diet modulates the gut microbiota and short chain fatty acids production in a salt-sensitive hypertension rat model. Nutrients 10:1154. doi: 10.3390/nu10091154, 30142973 PMC6164908

[ref5] Bjerrum A. Lindebjerg J. Andersen O. Fischer A. Lynge E. (2020). Long-term risk of colorectal cancer after screen-detected adenoma: Experiences from a Danish gFOBT-positive screening cohort. Int. J. Cancer 147, 940–947. doi: 10.1002/ijc.32850, 31894860

[ref6] Borrel G. Brugère J. F. Gribaldo S. Schmitz R. A. Moissl-Eichinger C. (2020). The host-associated archaeome. Nat. Rev. Microbiol. 18, 622–636. doi: 10.1038/s41579-020-0407-y, 32690877

[ref7] Brugère J.-F. Borrel G. Gaci N. Tottey W. O'Toole P. W. Malpuech-Brugère C. (2014). Archaebiotics: proposed therapeutic use of archaea to prevent trimethylaminuria and cardiovascular disease. Gut Microbes 5, 5–10. doi: 10.4161/gmic.26749, 24247281 PMC4049937

[ref8] Burnett-Hartman A. N. Lee J. K. Demb J. Gupta S. (2021). An update on the epidemiology, molecular characterization, diagnosis, and screening strategies for early-onset colorectal cancer. Gastroenterology 160, 1041–1049. doi: 10.1053/j.gastro.2020.12.068, 33417940 PMC8273929

[ref9] Buskermolen M. Cenin D. R. Helsingen L. M. Guyatt G. Vandvik P. O. Haug U. . (2019). Colorectal cancer screening with faecal immunochemical testing, sigmoidoscopy or colonoscopy: A microsimulation modelling study. BMJ 367:l5383. doi: 10.1136/bmj.l5383, 31578177 PMC6774435

[ref10] Castellarin M. Warren R. L. Freeman J. D. Dreolini L. Krzywinski M. Strauss J. . (2012). *Fusobacterium nucleatum* infection is prevalent in human colorectal carcinoma. Genome Res. 22, 299–306. doi: 10.1101/gr.126516.111, 22009989 PMC3266037

[ref11] Cavalcanti Y. W. Morse D. J. da Silva W. J. Del-Bel-Cury A. A. Wei X. Wilson M. . (2015). Virulence and pathogenicity of *Candida albicans* is enhanced in biofilms containing oral bacteria. Biofouling 31, 27–38. doi: 10.1080/08927014.2014.996143, 25574582

[ref12] Chen S. (2023). Ultrafast one-pass FASTQ data preprocessing, quality control, and deduplication using fastp. iMeta 2:e107. doi: 10.1002/imt2.107, 38868435 PMC10989850

[ref13] Coker O. O. Liu C. Wu W. K. K. Wong S. H. Jia W. Sung J. J. . (2022). Altered gut metabolites and microbiota interactions are implicated in colorectal carcinogenesis and can be non-invasive diagnostic biomarkers. Microbiome 10:35. doi: 10.1186/s40168-021-01208-5, 35189961 PMC8862353

[ref14] Drancourt M. Djemai K. Gouriet F. Grine G. Loukil A. Bedotto M. . (2021). *Methanobrevibacter smithii* archaemia in febrile patients with bacteremia, including those with endocarditis. Clin. Infect. Dis. 73, e2571–e2579. doi: 10.1093/cid/ciaa998, 32668457

[ref15] Flores C. O. Meyer J. R. Valverde S. Farr L. Weitz J. S. (2011). Statistical structure of host-phage interactions. Proc. Natl. Acad. Sci. 108, E288–E297. doi: 10.1073/pnas.1101595108, 21709225 PMC3136311

[ref16] Franzosa E. A. McIver L. J. Rahnavard G. Thompson L. R. Schirmer M. Weingart G. . (2018). Species-level functional profiling of metagenomes and metatranscriptomes. Nat. Methods 15, 962–968. doi: 10.1038/s41592-018-0176-y, 30377376 PMC6235447

[ref17] Gao R. Wang Z. Li H. Cao Z. Gao Z. Chen H. . (2020). Gut microbiota dysbiosis signature is associated with the colorectal carcinogenesis sequence and improves the diagnosis of colorectal lesions. J. Gastroenterol. Hepatol. 35, 2109–2121. doi: 10.1111/jgh.15077 32337748

[ref18] Hoarau G. Mukherjee P. K. Gower-Rousseau C. Hager C. Chandra J. Retuerto M. A. . (2016). Bacteriome and mycobiome interactions underscore microbial dysbiosis in familial Crohn’s disease. MBio 7, e01250–e01216. doi: 10.1128/mBio.01250-16, 27651359 PMC5030358

[ref19] Kandler O. König H. (1998). Cell wall polymers in Archaea (Archaebacteria). Cell. Mol. Life Sci. 54, 305–308. doi: 10.1007/s000180050156, 9614965 PMC11147200

[ref20] Khleborodova A. Gamboa-Tuz S. D. Ramos M. Segata N. Waldron L. Oh S. (2024). Lefser: implementation of metagenomic biomarker discovery tool, LEfSe, in R. Bioinformatics 40:btae707. doi: 10.1093/bioinformatics/btae707, 39585730 PMC11665633

[ref21] Kim M. Vogtmann E. Ahlquist D. A. Devens M. E. Kisiel J. B. Taylor W. R. . (2020). Fecal metabolomic signatures in colorectal adenoma patients are associated with gut microbiota and early events of colorectal cancer pathogenesis. MBio 11:e03186-19. doi: 10.1128/mbio.03186-19, 32071266 PMC7029137

[ref22] Kong C. Liang L. Liu G. Du L. Yang Y. Liu J. . (2023). Integrated metagenomic and metabolomic analysis reveals distinct gut-microbiome-derived phenotypes in early-onset colorectal cancer. Gut 72, 1129–1142. doi: 10.1136/gutjnl-2022-327156, 35953094

[ref23] Konishi Y. Okumura S. Matsumoto T. Itatani Y. Nishiyama T. Okazaki Y. . (2022). Development and evaluation of a colorectal cancer screening method using machine learning-based gut microbiota analysis. Cancer Med. 11, 3194–3206. doi: 10.1002/cam4.4671, 35318827 PMC9385600

[ref24] Kostic A. D. Gevers D. Pedamallu C. S. Michaud M. Duke F. Earl A. M. . (2012). Genomic analysis identifies association of *Fusobacterium* with colorectal carcinoma. Genome Res. 22, 292–298. doi: 10.1101/gr.126573.111, 22009990 PMC3266036

[ref25] Kumpitsch C. Fischmeister F. P. S. Mahnert A. Lackner S. Wilding M. Sturm C. . (2021). Reduced B12 uptake and increased gastrointestinal formate are associated with archaeome-mediated breath methane emission in humans. Microbiome 9:193. doi: 10.1186/s40168-021-01130-w, 34560884 PMC8464155

[ref26] Ladabaum U. Dominitz J. A. Kahi C. Schoen R. E. (2020). Strategies for colorectal cancer screening. Gastroenterology 158, 418–432. doi: 10.1053/j.gastro.2019.06.043 31394083

[ref27] Leslie A. (2002). The colorectal adenoma-carcinoma sequence. Br. J. Surg. 89, 845–860. doi: 10.1046/j.1365-2168.2002.02120.x, 12081733

[ref28] Li T. Coker O. O. Sun Y. Li S. Liu C. Lin Y. . (2025). Multi-cohort analysis reveals altered archaea in colorectal cancer fecal samples across populations. Gastroenterology 168, 525–538.e2. doi: 10.1053/j.gastro.2024.10.023, 39490771

[ref29] Li X. V. Leonardi I. Putzel G. G. Semon A. Fiers W. D. Kusakabe T. . (2022). Immune regulation by fungal strain diversity in inflammatory bowel disease. Nature 603, 672–678. doi: 10.1038/s41586-022-04502-w, 35296857 PMC9166917

[ref30] Li K. Zhang T. Ren H. Zhao W. Hong S. Ge Y. . (2023). Structural and physicochemical properties of bracken fern (*Pteridium aquilinum*) starch. Front. Nutr. 10:1201357. doi: 10.3389/fnut.2023.1201357, 37408989 PMC10318185

[ref31] Lin Y. Xie M. Lau H. C. H. Zeng R. Zhang R. Wang L. . (2024). Effects of gut microbiota on immune checkpoint inhibitors in multi-cancer and as microbial biomarkers for predicting therapeutic response. Med 6:100530. 39515321 10.1016/j.medj.2024.10.007

[ref32] Liu K. Yang X. Zeng M. Yuan Y. Sun J. He P. . (2021). The role of fecal *Fusobacterium nucleatum* and pks+ *Escherichia coli* as early diagnostic markers of colorectal cancer. Dis. Markers 2021:1171239. doi: 10.1155/2021/1171239 34853619 PMC8629656

[ref33] Luo S. Ru J. Mirzaei M. K. Xue J. Peng X. Ralser A. . (2023). Gut virome profiling identifies an association between temperate phages and colorectal cancer promoted by *Helicobacter pylori* infection. Gut Microbes 15:2257291. doi: 10.1080/19490976.2023.2257291, 37747149 PMC10578192

[ref34] Pereira M. B. Wallroth M. Jonsson V. Kristiansson E. (2018). Comparison of normalization methods for the analysis of metagenomic gene abundance data. BMC Genomics 19:274. doi: 10.1186/s12864-018-4637-6, 29678163 PMC5910605

[ref35] Petrovic Fabijan A. Lin R. C. Y. Ho J. Maddocks S. Ben Zakour N. L. Iredell J. R. . (2020). Safety of bacteriophage therapy in severe *Staphylococcus aureus* infection. Nat. Microbiol. 5, 465–472. doi: 10.1038/s41564-019-0634-z, 32066959

[ref36] Pimentel M. Lin H. C. Enayati P. van den Burg B. Lee H.-R. Chen J. H. . (2006). Methane, a gas produced by enteric bacteria, slows intestinal transit and augments small intestinal contractile activity. Am. J. Physiol. Gastrointest. Liver Physiol. 290, G1089–G1095. doi: 10.1152/ajpgi.00574.2004, 16293652

[ref37] Richard M. L. Sokol H. (2019). The gut mycobiota: insights into analysis, environmental interactions and role in gastrointestinal diseases. Nat. Rev. Gastroenterol. Hepatol. 16, 331–345. doi: 10.1038/s41575-019-0121-2, 30824884

[ref38] Samuel B. S. Gordon J. I. (2006). A humanized gnotobiotic mouse model of host-archaeal-bacterial mutualism. Proc. Natl. Acad. Sci. 103, 10011–10016. doi: 10.1073/pnas.0602187103, 16782812 PMC1479766

[ref39] Samuel B. S. Hansen E. E. Manchester J. K. Coutinho P. M. Henrissat B. Fulton R. . (2007). Genomic and metabolic adaptations of *Methanobrevibacter smithii* to the human gut. Proc. Natl. Acad. Sci. 104, 10643–10648. doi: 10.1073/pnas.0704189104, 17563350 PMC1890564

[ref40] Singh P. Duehren S. Katon J. Rangan V. Ballou S. Patel R. . (2020). Breath methane does not correlate with constipation severity or bloating in patients with constipation. J. Clin. Gastroenterol. 54, 365–369. doi: 10.1097/MCG.0000000000001239, 31306344

[ref41] Sogodogo E. Drancourt M. Grine G. (2019). Methanogens as emerging pathogens in anaerobic abscesses. Eur. J. Clin. Microbiol. Infect. Dis. 38, 811–818. doi: 10.1007/s10096-019-03510-5, 30796545

[ref42] Sokol H. Leducq V. Aschard H. Pham H. P. Jegou S. Landman C. . (2017). Fungal microbiota dysbiosis in IBD. Gut 66, 1039–1048. doi: 10.1136/gutjnl-2015-310746, 26843508 PMC5532459

[ref43] Wang L.-W. Ruan H. Wang B. M. Qin Y. Zhong W. L. (2023). Microbiota regulation in constipation and colorectal cancer. World J. Gastrointest. Oncol. 15, 776–786. doi: 10.4251/wjgo.v15.i5.776, 37275451 PMC10237018

[ref44] Wong S. H. Yu J. (2019). Gut microbiota in colorectal cancer: mechanisms of action and clinical applications. Nat. Rev. Gastroenterol. Hepatol. 16, 690–704. doi: 10.1038/s41575-019-0209-8, 31554963

[ref45] Yachida S. Mizutani S. Shiroma H. Shiba S. Nakajima T. Sakamoto T. . (2019). Metagenomic and metabolomic analyses reveal distinct stage-specific phenotypes of the gut microbiota in colorectal cancer. Nat. Med. 25, 968–976. doi: 10.1038/s41591-019-0458-7, 31171880

[ref46] Yang Y. Weng W. Peng J. Hong L. Yang L. Toiyama Y. . (2017). *Fusobacterium nucleatum* increases proliferation of colorectal cancer cells and tumor development in mice by activating Toll-like receptor 4 signaling to nuclear factor-kB, and up-regulating expression of microRNA-21. Gastroenterology 152, 851–866. doi: 10.1053/j.gastro.2016.11.018, 27876571 PMC5555435

[ref47] Yao Y. Ni H. Wang X. Xu Q. Zhang J. Jiang L. . (2021). A new biomarker of fecal bacteria for non-invasive diagnosis of colorectal cancer. Front. Cell. Infect. Microbiol. 11:744049. doi: 10.3389/fcimb.2021.744049, 34976850 PMC8719628

[ref48] Zeller G. Tap J. Voigt A. Y. Sunagawa S. Kultima J. R. Costea P. I. . (2014). Potential of fecal microbiota for early-stage detection of colorectal cancer. Mol. Syst. Biol. 10:766. doi: 10.15252/msb.20145645, 25432777 PMC4299606

[ref49] Zhang Z. Chen Y. Pan X. Li P. Ren Z. Wang X. . (2024). IL-1β mediates *Candida tropicalis*-induced immunosuppressive function of MDSCs to foster colorectal cancer. Cell Commun. Signal. 22:408. doi: 10.1186/s12964-024-01771-y, 39164774 PMC11337875

[ref50] Zhao Y. Zhu M. Ling Y. Zhao Y. Lu X. Chu B. . (2025). A DNA nanopatch-bacteriophage system targeting *Streptococcus gallolyticus* for inflammatory bowel disease treatment and colorectal cancer prevention. Adv. Mater. 37:e2417334. doi: 10.1002/adma.202417334 39924920

[ref51] Zhou B. Wang C. Putzel G. Hu J. Liu M. Wu F. . (2024). An integrated strain-level analytic pipeline utilizing longitudinal metagenomic data. Microbiol. Spectrum 12:e0143124. doi: 10.1128/spectrum.01431-24, 39311770 PMC11542597

[ref52] Zhu Y. Shi T. Lu X. Xu Z. Qu J. Zhang Z. . (2021). Fungal-induced glycolysis in macrophages promotes colon cancer by enhancing innate lymphoid cell secretion of IL-22. EMBO J. 40:e105320. doi: 10.15252/embj.2020105320, 33591591 PMC8167358

[ref53] Zillig W. (1991). Comparative biochemistry of archaea and bacteria. Curr. Opin. Genet. Dev. 1, 544–551. doi: 10.1016/S0959-437X(05)80206-0, 1822288

[ref54] Zwezerijnen-Jiwa F. H. Sivov H. Paizs P. Zafeiropoulou K. Kinross J. (2023). A systematic review of microbiome-derived biomarkers for early colorectal cancer detection. Neoplasia 36:100868. doi: 10.1016/j.neo.2022.100868, 36566591 PMC9804137

