# Supplementary figures and images for: Unveiling the unique gut microbial signatures in colorectal adenomas: establishment and validation of a cross-kingdom microbiome predictive model
Source: Front Microbiol. 2026 Jun 16;17:1854806. doi: 10.3389/fmicb.2026.1854806 (PMC13314950; doi:10.3389/fmicb.2026.1854806)

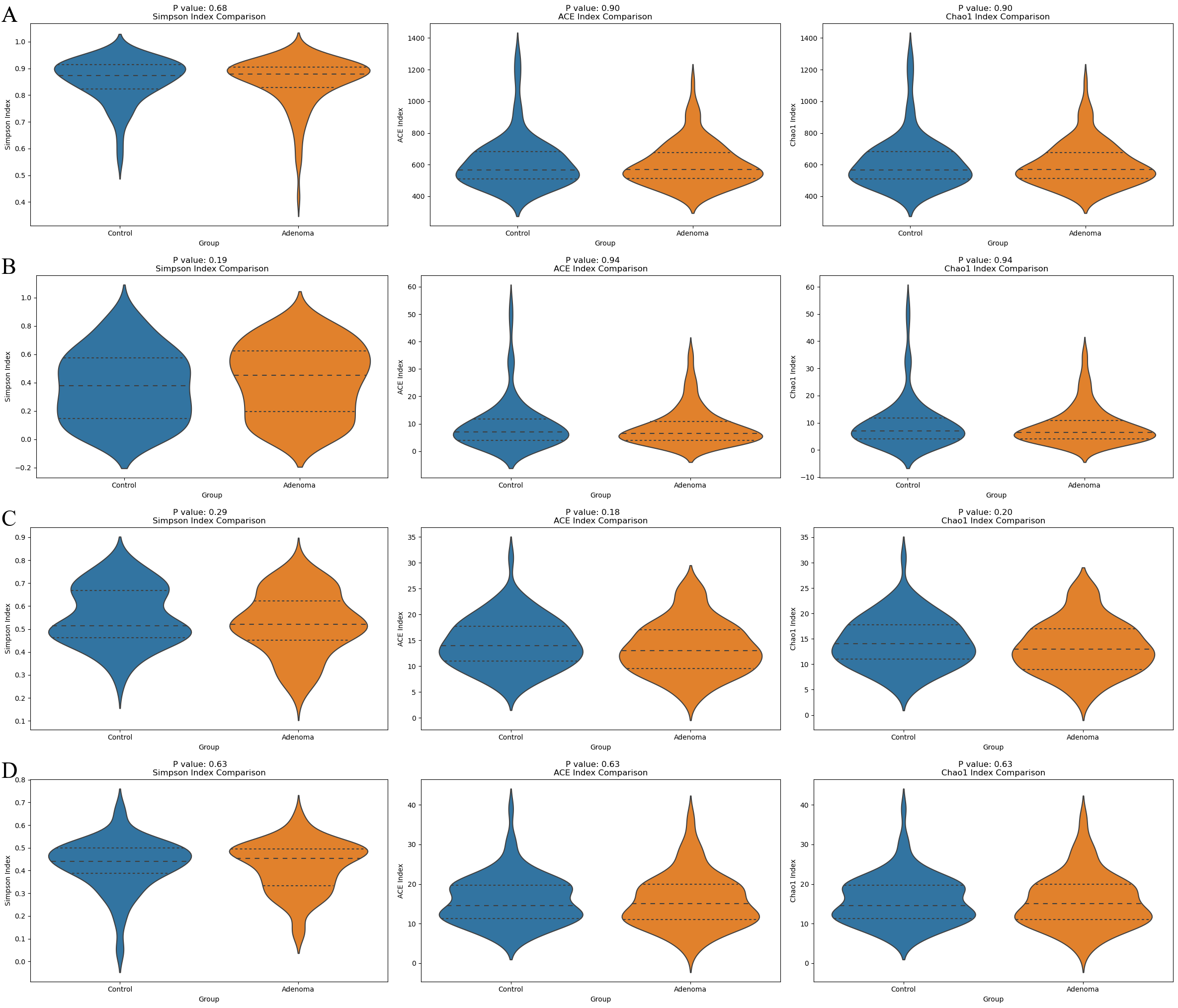

Supplement: SUPPLEMENTARY FIGURE S1 — (A) Violin plots of α-diversity at the bacterial species level. (B) Violin plots of α-diversity at the fungal species level. (C) Violin plots of α-diversity at the archaeal species level. (D) Violin plots of α-diversity at the viral species level. Statistical differences were compared using the Mann-Whitney U test. [file Image_1.TIF]

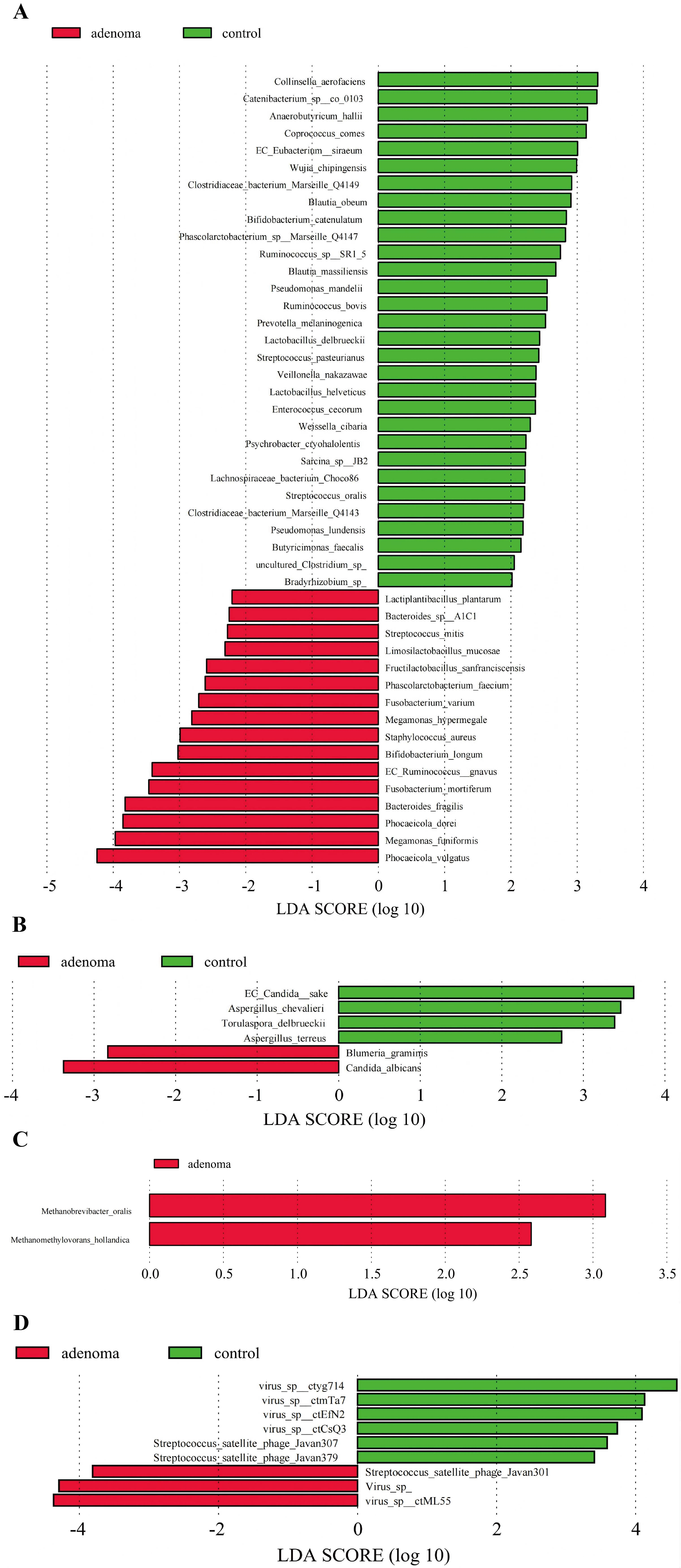

Supplement: SUPPLEMENTARY FIGURE S2 — Lefse analysis of 112 high-altitude population samples: (A) Lefse analysis of bacteria; (B) Lefse analysis of fungi; (C) Lefse analysis of archaea; (D) Lefse analysis of viruses. [file Image_2.TIFF]
